# A survey on deep learning for drug-target binding prediction: models, benchmarks, evaluation, and case studies

**DOI:** 10.1093/bib/bbaf491

**Published:** 2025-09-22

**Authors:** Kusal Debnath, Pratip Rana, Preetam Ghosh

**Affiliations:** Department of Computer Science, Virginia Commonwealth University, Richmond, VA 23284, United States; Department of Computer Science, Old Dominion University, Norfolk, VA 23529, United States; Department of Computer Science, Virginia Commonwealth University, Richmond, VA 23284, United States

**Keywords:** artificial intelligence, deep learning, drug–target interaction, drug-target affinity, cancer drug discovery

## Abstract

Conventional drug discovery is expensive, time-consuming, and prone to failure. Artificial intelligence has become a potent substitute over the last decade, providing strong answers to challenging biological issues in this field. Among these difficulties, drug-target binding (DTB) is a key component of drug discovery techniques. In this context, drug-target affinity and drug–target interaction are complementary and essential frameworks that work together to improve our comprehension of DTB dynamics. In this work, we thoroughly analyze the most recent deep learning models, popular benchmark datasets, and assessment metrics for DTB prediction. We look at the paradigm shift in the development of drug discovery research since researchers started using deep learning as a potent tool for DTB prediction. In particular, we examine how methodologies have evolved, starting with early heterogeneous network-based approaches, progressing to graph-based approaches that were widely accepted, followed by modern attention-based architectures, and finally, the most recent multimodal approaches. We also provide case studies utilizing an extensive compound library against specific protein targets implicated in critical cancer pathways to demonstrate the usefulness of these approaches. In addition to summarizing the latest developments in DTB prediction models, this review also identifies their drawbacks. It also highlights the outlook for the DTB prediction domain and future research directions. Combined, these studies present a more comprehensive view of how deep learning offers a quantitative framework for researching drug-target relationships, speeding up the identification of new drug candidates and making it easier to identify possible DTBs.

## Introduction

Drug-target binding (DTB) prediction plays a pivotal role in drug discovery. DTB analysis unfolds the possible interactions of drug compounds with target proteins crucial in several biological pathways involving various diseases. Traditional approaches for identifying DTBs demand a heavy investment in time and cost. Recent advances in computational methods, particularly artificial intelligence (AI)-based approaches, showcased remarkable progress in DTB prediction. These approaches act as reliable alternatives, diminishing the constraints tied to traditional methods and offering better accuracy.

In early developments, statistics and classical machine learning methods were predominant approaches, leveraging manually curated descriptors or features of drugs and targets. However, a significant challenge of these methods is that they depend solely on available clinical data for analysis. In addition, there are challenges in manually selecting features for model training, as it requires in-depth knowledge of pharmacodynamics concepts. Also, some of these methods require iterative analysis through standard statistical methods, which are susceptible to errors. In contrast, deep learning gained popularity because of its ability to handle large datasets, better performance, and ability to learn intricate relationships between input data and output.

The last decade witnessed a surge in deep learning-driven DTB prediction methodologies, fueled by the widespread availability of data resources hosting information related to molecular entities and biological networks related to specific cell lines and diseases. In addition, improved computational power facilitated the development of more sophisticated deep-learning algorithms. Such deep learning models’ ability to handle large amounts of data and learn complex non-linear relations from them is a crucial aspect of modern DTB research.

Deep learning-based approaches can extract complex features and their inter-relations through a network of artificial neurons, diminishing the challenge of manual selection of features important for prediction. The availability of structural and functional information on compounds and proteins allows the application of such approaches in modeling DTB prediction problems. Early approaches in modeling DTB prediction task leverage simpler feature extraction methods using convolutional neural networks (CNNs) and recurrent neural networks from one-dimensional sequential information (e.g. SMILES [1], SMARTS [2], and SMIRKS [3]) of drugs and targets (e.g. amino acid sequences). Although these approaches showed superior results to earlier statistical and machine learning-based methods, they had some significant limitations. Firstly, they addressed drugs and proteins in their native primary-structural forms, often ignoring their three-dimensional configurations consisting of bond lengths, bond angles, inter-atomic distances, and scaffolds. In addition, information on specific binding pockets is often missing in these representations, limiting the modeling of chemistry-informed binding inside the human system.

Later developments using the primary representations of drugs and compounds utilized more sophisticated feature extraction methods such as graph-based, attention-based, and hybrid approaches. Though working with the exact one-dimensional representations, graph-based methods further represented the molecules in higher-dimensional graphs considering the positional aspects of constituent atoms. Attention-based approaches discussed concepts such as multi-headed attention, mutual learning, and feature aggregation for extracting more complex features relevant to modeling DTB prediction tasks, providing better results than previous methods. Some hybrid methods also demonstrate significant results in DTB prediction, incorporating information related to drugs and targets encompassing spatial chemical environments, molecular substructure-specific modeling, similarity accumulation, cross-attention, and molecular augmentation.

Recent developments in natural-language-based methods paved the way for representing DTB prediction as a hybrid-natural language-based problem, extracting semantic features from the drug and target structures. Developing domain-specific large language model (LLM) derivatives from the established ones (e.g. ChemBERTa [4] and ProtBERT [5] derived from original BERT [6] model) is an active research area in the field of drug discovery. These language models facilitate drug discovery research by providing crucial semantic information from chemical structures. Generated embeddings are combined with previously established approaches for DTB prediction, including graph-based and attention-based methods that aid in a better understanding of the importance of features and provide better prediction results.

**Figure 1:**
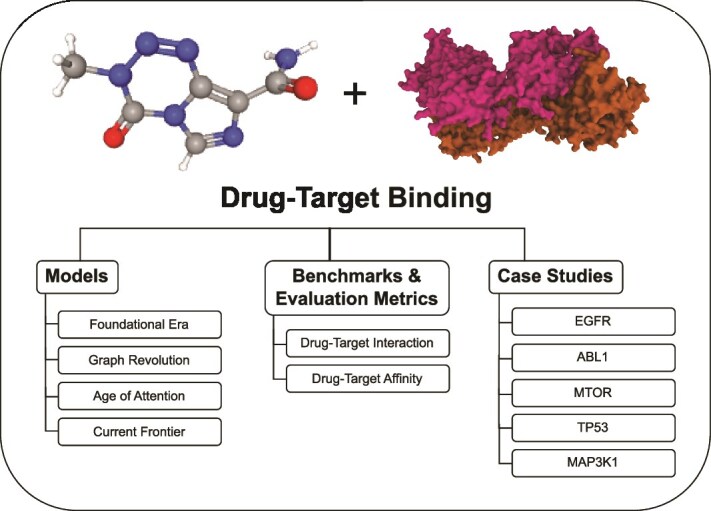
Graphical representation of the essential components discussed through systematic survey.

In this work, we conducted an evaluation-based survey for DTB prediction approaches. In the Background section, we discussed the early attempts and how the continuous development led to the most recent modern attempts in drug-target affinity (DTA) predictions. In the methodology section, we discuss the model selection procedure, summarize the selected models, discuss standard datasets used for benchmarking, and evaluate the results. In addition, we conducted multiple case studies to predict DTB in cancer, using crucial proteins in key biological pathways associated with various types of cancer. Finally, we briefly discussed the general research scenario for deep learning-based DTB prediction. In summary, this work offers valuable insights into the current research landscape of deep learning-driven DTB prediction, which will support researchers in advancing groundbreaking findings in this field. A comprehensive overview of the evaluation workflow is represented in Fig. 1.

## Background

The quest for potential drug targets and identifying suitable drugs for targets has always been one of the central topics in targeted cancer therapy. Advancements in computational technologies have only facilitated the extent of this quest. Since the inception of deep learning, the research in the virtual screening of drugs has advanced considerably. Nevertheless, the earlier developments remain highly relevant, as they are crucial in laying the foundation for the current progress. This section discusses the early discoveries in the DTB domain and how they have inspired modern deep-learning approaches. This section thus can be divided into two key stages—the pre-deep learning era and the post-deep learning era.

### Pre-deep learning era

At this stage, researchers primarily relied on the extensive clinical data on the topological and therapeutic properties of drugs and physiological interactions related to target proteins available in various databases. In an earlier study, Yıldırım *et al.* [7] built a bipartite graph containing FDA-approved drugs and proteins linked by drug-target binary associations. Clusters of drugs were created according to the anatomy, therapeutic properties, and chemical configuration of each drug. This network emphasized the prevalence of “follow-on” drugs that target already targeted proteins. The goal was to integrate the principles of network biology and the knowledge of drug–target interactions (DTIs) to analyze drug-target networks in cells and their mutual interactions with disease gene products. Bleakley *et al.* [8] used a supervised inference approach called bipartite local models, where target proteins for a given drug and target drugs for a given protein were predicted independently for each drug-target pair. Mei *et al.* [9] presented a method called neighbor-based interaction-profile inferring and integrated it into the existing bipartite local model to handle unknown DTIs. The interaction profile is treated as label information. It is used for model learning of new candidates, crucial in finding targets for new drug candidates and drugs for new target candidate proteins.

Yamanishi *et al.* [10, 11] found that DTIs correlate more with pharmacological effect similarity than chemical structure similarity. They emphasized mapping the pharmacological effects of chemical, genomic, and pharmacological data of given compounds. Iorio *et al.* [12] utilized similarities among gene expression profiles following drug treatment for multiple cell lines and dosages to get estimates on drug effects and mode of action (MoA). Drug compounds are clustered based on their MoA or targeting a specific biological pathway, thus assessing the usability of multiple drugs for a common disease. Perlman *et al.* [13] introduced a novel approach for scoring the drug-gene association by combining the drug-drug and gene-gene similarity measures through a logistic regression component to integrate multiple association scores to give an estimate of the final association score, emphasizing the importance of heterogeneous information integration for DTI prediction. Mizutani *et al.* [14] introduced a novel approach combining the drug-protein interactions and side effects linking the molecular and side effects scale of drug actions. The work discusses the analysis of the co-occurrence of drugs in protein-binding profiles and side-effect profiles, proven to be effective for predicting possible side-effects of new drug candidates based on their protein-binding profiles. Campillos *et al.* [15] utilized phenotypic side-effect similarities to infer potential shared targets between two drugs, discovering novel DTIs. However, one significant downside of this method is the manual inference of the shared targets between drug pairs, which can be a time-consuming and error-prone process. In addition, there exists a degree of uncertainty in accurately associating a given target with a specific side effect. Sirota *et al.* [16] demonstrated the importance of molecular signatures in drug-disease pairs for DTI prediction. They integrated gene-expression measurements from several diseases and drugs, emphasizing the fact that drugs exerting similar gene expressions may have some degree of similarity. Chen *et al.* [17] utilized a heterogeneous network approach, combining protein-protein similarity network, drug-drug similarity network, and known DTI network with further integration with a random walk. They emphasized the hypothesis that similar drugs often target similar target proteins.

Li *et al.* [18] introduced a web tool named TarFishDock. The reverse docking methodology is utilized for ligand-protein docking to search for potential protein targets for specific drugs by screening protein databases. Kinnings *et al.* [19] addressed the limitations of docking scoring functions in assigning bond weights correctly and the inaccuracy in mapping the inter-dependency of noncovalent bonds while scoring. They introduced an improved scoring function by training support vector machines on IC50 values of BindingDB [20] dataset and directory of useful decoys (DUD) dataset [21] utilizing the individual energy terms retrieved from molecular docking along with the known binding affinity values from high throughput screening experiments. Shaikh *et al.* [22] propose proteochemometric models for enhanced DTI prediction, emphasizing the importance of negative instances in the prediction task. Moreover, a novel fingerprint-based approach was demonstrated to develop the applicability domain of the proposed models.

Van Laarhoven *et al.* [23] demonstrated that machine learning algorithms can accurately predict DTIs using limited information. They introduced a kernel-based approach known as Gaussian interaction profile (GIP), which uses the interaction profiles of drug-target networks and a simple classifier called Regularized Least Squares to predict these interactions. In a later work, they introduced a weighted nearest neighbor (WNN) approach [24] to predict unknown DTIs, where an interaction score profile is constructed for a new drug based on the chemical and interaction information similarity of the existing drug compounds in the dataset. Further, they integrated GIP and WNN and called it WNN-GIP. Gonen *et al.* [25] used a novel approach called Bayesian formulation, combining dimensionality reduction, matrix factorization, and binary classification to predict DTIs. Similarity analysis was performed for drug compounds based on their chemical structures. For target proteins, corresponding genomic similarities were used to project the drugs and proteins in a unified subspace for prediction. Wang *et al.* [26] demonstrated DTI prediction as a two-layer graphical model known as a restricted Boltzmann Machine (RBM). Here, DTIs are represented as multi-dimensional networks. A practical learning algorithm called Contrastive Divergence is used here, and it utilizes the probability distribution over input data to train the RBM. Cobanoglu *et al.* [27] proposed a collaborative filtering algorithm called probabilistic matrix factorization (PMF), which can predict DTIs by analyzing large interaction networks without additional similarity information.

While the methods mentioned above showed some success in predicting DTIs, they were hindered by several limitations, such as a) solely depending on experimental clinical data for analysis can lead to challenges due to the ambiguity of data across databases; b) the manual selection of significant features among entities for prediction is a meticulous process; and c) iterative statistical analyses, while crucial, are susceptible to errors in calculations. In response to these challenges, researchers have turned to deep learning-based methods for DTI prediction, offering a more robust and practical approach.

### Post-deep learning era

Since its inception, deep learning has provided promising approaches to automate feature extraction by learning salient representations from raw biological data. In this section, we scrutinize the chronological progression of deep learning applications enhancing DTB predictions, from initial network-based and sequence-based approaches through the revolution of graph-based modeling, and finally, to the current transformer-based and multi-modal fusion approaches.

#### The foundational era: network integration and sequence-based methods

This phase marked the pivot from traditional, feature engineering-based machine learning approaches toward automated feature extraction-based deep learning approaches. This era majorly involves the exploitation of heterogeneous network-based approaches. Utilization of 1D biological sequences became mainstream. Additionally, due to the advent of large-scale experimental bioactivity assays, this period witnessed the clear bifurcation of DTI classification and DTA regression tasks; therefore, a separate set of evaluation approaches, metrics, and benchmarks emerged.

Luo *et al.* addressed the limitation that existing DTI methods were primarily designed for homogeneous networks or simple bipartite models that got troubled handling the noisy, incomplete, and high-dimensional biological data in their proposed approach called DTINet [28]. DTINet revolutionized this approach by integrating diverse heterogeneous data sources into a unified network. It learned low-dimensional vector representations that capture the topological properties of nodes (drugs and proteins). This approach aided in finding an optimal projection from drug space to protein space, such that feature vectors of drugs are geometrically close to the feature vectors of their known interacting proteins. Later, DeepDR [29] used a more sophisticated multi-modal deep autoencoder in their model architecture to enhance the non-linear relationships capturing among individual networks in the heterogeneous network setup. It provides pharmacological interpretability and visualizable vector representations of network vertices.

Öztürk *et al.* introduced DeepDTA [30] and pioneered in framing the DTI prediction as a regression task with binding affinity values instead of a binary classification task. They simplified the 1D representation of drugs and targets, diminishing reliance on 3D drug-protein complexes. They adopted a CNN-based automatic feature learning approach from raw data instead of manual feature engineering. Instead of indirect learning from network-based/similarity-based approaches, they attempted direct learning from input drug and protein structural data using vectorization of the string-based data.

Lee *et al.* proposed DeepConvDTI [31], which uses CNN-based feature extraction from raw protein sequences to address the limitation of pre-computed descriptors (e.g. CTD), which were unable to capture crucial local features and binding-site specific features.

While graph-based approaches were proven to outperform sequence-based approaches, Wang *et al.* DeepDTAF [32] emphasized utilizing the secondary structural features and binding pocket information and outperformed models like Pafnucy in their proposed model named DeepDTAF [32]. It combines binding pocket features (local) with entire protein features (global). In a similar work, Dou *et al.* later introduced a more granular, fragment-oriented framework called BCM-DTI [33] that extracts diverse fragment types (branch chain, common substructure, motif/fragments) leveraging biological knowledge.

To mitigate the limitation of the requirement of 3D structural data, which also hinders scaling due to issues like low quality and computational bottleneck, Thafar *et al.* proposed Affinity2Vec [34], which transformed the DTI problem as a graph-based regression problem utilizing a heterogeneous graph network.

While the models above often tried to bypass the dependency on 3D structural data, Zheng *et al.* proposed OnionNet [35] leveraging 3D compound-protein complex data from the PDBBind dataset (v2016) to work toward physics-based atomic-level interaction prediction approach constructing multiple sequentially distant shells to capture both short-range (van der Waals) and long-range (electrostatic) interactions. This work thus laid the foundation for the current rotation-invariant molecular representation in de novo drug design while outperforming previous state-of-the-art physics-based 3D binding approaches like Pafnucy [36] and KDeep [37].

Recently, researchers explored the impact of similarity features, lower-dimensional inductive embeddings, structural topology, semantic graphs, and contrastive learning-oriented approaches for means of heterogeneous network-based binding prediction [38–40].

#### The graph revolution: embracing molecular topology

In this phase, researchers started representing drugs as 2D graphs, which better showcase the structural topology instead of 1D strings. Future studies prove that representing drugs as graphs is more effective than as strings. This conceptual drift laid foundational stones for representing macromolecules like proteins as graphs. At a later stage, with the integration of attention mechanisms, graph-based approaches became more robust.

Existing sequence-based methods represented drugs as 1D strings, unable to capture crucial topological features like inter-atomic bond length and angles. Nguyen *et al.* pioneered in representing drugs as 2D molecular graphs by introducing GraphDTA [41], where nodes of the graphs represent atoms and edges represent bonds. They used RDKit [42] to extract atomic features (atom symbol, number of adjacent atoms/hydrogens, implicit value, aromatic structure, etc.), which are eventually used as the node features. Similarly, Tran *et al.* proposed DeepNC [43], representing drugs as graphs and employing various graph neural network (GNN) algorithms to learn their chemical and structural characteristics. Later, Voitsitskyi *et al.* proposed 3DProtDTA [44] leveraging AlphaFold’s [45] protein structure predictions to generate residue-level protein graphs, thereby providing abundant structural data and preserving connectivity and 3D arrangement information. This model performed equally well when evaluated on cold targets containing tyrosine-protein kinases and serine/threonine protein kinases.

Jiang *et al.* went forward with not only representing biological entities as graphs, they also encoded protein structures as graphs and introduced DGraphDTA [46]. They utilized contact map prediction to capture protein spatial structure from raw sequences. These contact maps are then used as graph edges, while the amino acids are used as nodes.

By incorporating the weight-assigning ability of the attention mechanism in MHSADTI [47], Cheng *et al.* utilized a graph attention network (GAT) for drugs to reduce the effect of noisy connections within the nodes of the drug graph structure. Visualization of high-scoring attention weights provides interpretability of the approach. Similarly, Yu *et al.* incorporated multi-scale encoders for both drugs and proteins in an interpretable approach called MSFFDTA [48], developing a Selective cross-attention mechanism to filter out trivial interactions and focusing on crucial drug-protein substructure pairs. Recently, Wang *et al.* proposed WDGBANDTI [49] where they used a deep graph convolutional network (GCN) to extract rich atomic and sub-structural drug information, a CNN for protein features, and a Bilinear Attention Network to learn and visualize local sub-structural interactions explicitly providing interpretability.

Zhang *et al.* proposed DeepMGT-DTI [50] to discuss the fragile nature of SMILES sequences, as a slight change in SMILES structure can drastically alter molecular conformation. In addition, precise inter-atomic interaction estimation was often impossible from only sequence information. Thus, they proposed a multilayer graph fusion approach using a transformer network to accumulate information from different hidden layers of the graph convolution network. Moreover, they implemented a multi-headed attention architecture (four and six heads) to enhance edge feature learning, which was often missing in previous graph-based approaches. He *et al.* proposed NHGNN-DTA [51], a hybrid GNN that combines sequence-based feature generation with graph-level information interaction, including a unique central node to connect drug and protein graphs. They performed three types of splitting for cold-start scenarios: (i) cold-drug, (ii) cold-target, and (iii) cold-drug-target. For interpretability analysis, they provided attention-weight visualization of the final output.

Wang *et al.* introduced MSN-DTA [52] to address the limitations of current GNN-based DTA models that poorly extract drug molecular substructure information and rely on insufficient manually configured node features, which hinders generalization for similar structures, by introducing a multi-scale graph isomorphic network to extract multi-level structural information. To estimate the interpretability of DTI, they provided attention-weight visualization of protein binding sites. Similarly, Luo *et al.* used a hybrid GATv2-GCN and three-layer GCN in their proposed approach named GS-DTA [53] for a comprehensive, hierarchical drug feature extraction strategy with dynamic attention and global context, and by combining CNN, Bidirectional Long Short-Term Memory (BiLSTM), and Transformer for multi-level (local, contextual, and global) protein feature extraction.

Wang *et al.* proposed DHGT-DTI [54] to address the limitations of heterogeneous network methods that often fail to fully integrate both local and global network information simultaneously and struggle with generalization to new drug-target pairs by proposing a novel dual-view heterogeneous network approach that comprehensively captures network structural information from both local [using heterogeneous Graph Sample and Aggregate (GraphSAGE) for neighbor features] and global (using Graph Transformer with residual connections for higher-order meta-path features). To assess the generalizability of this approach, they used 1:4 cold-splitting. Similarly, Zhao *et al.* utilized meta-paths to represent regulatory mechanisms, constructing regulation graphs through random-walk sampling in an approach named RGLDR [55].

Wu *et al.* proposed AttentionMGT-DTA [56], which utilizes AlphaFold2-predicted protein structures to construct protein pocket graphs with rich spatial information to mitigate the limitation of 2D protein contact maps used in previous graph-based methods, which are unable to describe higher-dimensional protein structures accurately. In addition, they used a cross-attention module to fuse 1D sequence and 3D graph protein features. It provides high interpretability through its visualized attention matrices. Fusing biological and chemical property information in the model architecture and utilizing pre-trained embeddings enabled this approach to handle cold-start scenarios efficiently.

Zhang *et al.* proposed a graph dilated convolution strategy called GDilatedDTA [57] for improved feature extraction from indirect neighborhoods, integrating a multilayer residual connection network for local chemical information from SMILES and utilizing BiLSTM for target sequences, thereby enhancing interpretability, predictive accuracy and robustness in cold-start scenarios.

iGRLDTI [58], proposed by Zhao *et al.*, addresses the over-smoothing issue prevalent in GNN-based DTI models, which reduces the discriminative ability of learned representations by adaptively determining the node-specific propagation depth for each biomolecule using a node-dependent local smoothing strategy.

Huang *et al.* integrated GNNs with a self-supervised invariant feature learning module in their proposed approach called GFLearn [59] to extract robust and generalizable features and reduce dependence on specific feature patterns. Integrating one-dimensional and three-dimensional embedding using cross-attention has proven to be effective in the cold-start scenarios in this approach.

#### The age of attention: influence of natural language processing

In this era, studies were conducted to complement the topological feature extraction using graph-based approaches with promising attention-based approaches for focusing on extracting essential features. In addition, pre-trained language models (PLMs) started taking center stage in diverse learning scenarios, namely imbalanced data, data scarcity, and label bias, eventually providing interpretability for the models.

DrugVQA [60] pointed out the limitation of 1D protein sequences representing 3D spatial features crucial for DTB. On the other hand, the scarce nature of co-crystallized 3D protein data leads to sparse feature generation. Hence, they figured out a middle ground by utilizing 2D protein distance maps to mitigate the limitations above. They represented the DTI as a visual question-answering problem, where protein distance maps are the “images” and drug SMILES are the “questions.” Thus diminishing the reliability of 3D protein structures. Multi-headed attention is used to identify binding pocket regions in protein sequences and crucial molecular fragments within drug structures, while attention visualization is used to showcase interpretability.

TransformerCPI [61] addressed the hidden ligand bias in the DUD-E dataset that leads to learning only ligand patterns instead of true protein–ligand interactions and emphasized appropriate data for better generalizability. It used curated G-Protein Couple Receptor (GPCR) and Kinase datasets from pre-existing datasets, where each ligand appears in positive and negative classes. It performed rigorous tests where ligands appeared only in one class during training but in opposite classes during testing. Finally, it used a self-attention mechanism for dynamic feature extraction instead of memorizing ligand patterns. The interpretability of this approach was estimated by mapping attention weights back to protein sequences and compound molecules.

DeepCDA [62] highlighted a fundamental statistical misconception that training data and test data come from the same distribution. Training and test data may come from entirely different distributions in real-world scenarios. Thus, they proposed an effective adversarial learning method—Adversarial Discriminative Domain Adaptation. This method makes the model learn from a test domain feature encoder using adversarial domain adaptation between training and unlabeled test data. The adapted test encoder is then applied to predict binding affinity in the cold-start scenario. This approach utilizes two-sided attention to calculate pairwise compound-protein interaction.

MolTrans [63] emphasized that not the entire drug/protein structure participates in interactions; instead, specific repetitive regions within drug/targets are responsible for the interaction. They called these regions Frequent Consecutive Sequence (FCS), which were mined from the overall structure using a hierarchical decomposition mechanism. Drugs/proteins are tokenized into their most minor units, then sequentially combined to form frequently occurring sub-sequences. They used transformer encoders to extract contextual relationships within the sub-sequences, providing interpretability.

Observing the sole dependence on molecular features only and neglecting the interaction features in previous DTI prediction tasks, DeepFusion [64] adopted a multiscale feature fusion approach to combine global structural similarity features with local sub-structure features (carrying on the idea of FCS in MolTrans) utilizing a two-channel architecture. Similarly, GIFDTI combines CNN and transformer architecture to extract global molecular and intermolecular features (IIF module). To effectively explain the validity and interpretability of the IIF module, they map the intermolecular interaction scores of proteins calculated from the IIF module onto the 3D structure and protein sequence. IHDFN-DTI [65] introduces a hybrid deep feature extraction module for proteins that captures multi-level information via dynamic encoding and DIFF Transformer and fuses them with StarNet. Even though the approach achieves interpretability through attention focusing, the protein sequence embeddings were generated randomly, resulting in an embedding matrix that lacked biological context.

MONN [66] pointed out the issue of interpretability of attention mechanisms in DTI. It states that attention mechanisms only show pseudo-interaction among drug-target pairs by showing mere correlations among them instead of capturing actual non-covalent interactions. It utilizes a dual-attention network (DAN) for monitoring individual DTI pairs. It combines compound and protein features using predicted pairwise interactions as links as part of a multi-objective framework for simultaneously predicting binding affinities and non-covalent interactions among drug-target pairs.

HyperAttentionDTI [67] emphasized the shallow DTI modeling nature of the existing attention-based approaches and proposed a superior approach of modeling semantic inter-dependencies in both spatial and channel dimensions to capture more granular atom-amino acid binding by assigning pairwise attention vectors. Similarly, GraphsformerCPI [68] integrates semantic and spatial structural features of compounds and proteins using a structure-enhanced self-attention mechanism, which provides interpretability. SMFF-DTA [69] represents structural information and physicochemical properties of drugs and targets sequentially with innovative encoding and employing multiple attention blocks.

While approaches like BiCOMP-DTA [70] propose a unified, complementary protein encoding measure (BiComp) to address the computational overheads, complexity, and reliance on multiple data sources of existing DTA methods and showcase consistent results in cold-start scenario, MGDTI [71] employs a meta-learning framework that rapidly adapts to unseen tasks, incorporating drug-drug and target-target structural similarity to mitigate data scarcity.

BINDTI [72] provides an end-to-end framework that encodes drug features using GCNs and protein features with a mixed ACmix model and fuses these features through a bi-directional Intention network. FragXsiteDTI [73] leverages information-rich representations from drug molecule fragments and protein pockets and employs a transformer-based architecture with a learnable latent array that acts as a mediator for seamless and insightful information translation. It demonstrates high interpretability by attention score visualization for binding sites.

PLMs are pre-trained on large amounts of data. Thus, they are most suitable for small-sample learning and often perform better in cold-start scenarios. DTI-BERT [74] utilizes pre-trained BERT (ProtBert) for context-aware protein sequence features and Discrete Wavelet Transform for drug molecular fingerprints. DTI-LM [75] leverages PLM for sequence encoding and enhancing these representations with neighborhood information through GATs. They discussed the usefulness of pre-trained models, especially for proteins, in handling cold-start problems. LANTERN [76] integrates LLMs and Transformer-based architectures to generate high-quality, context-aware embeddings for drug and protein sequences and employs a Transformer-based fusion mechanism that enhances scalability, predictive accuracy, and generalizability without requiring 3D structural data. G-K BertDTA [77] proposed a novel framework that integrates protein features using a redesigned DenseSENet (DenseNet with Squeeze-and-Excitation blocks), molecular structural information using an improved Graph Isomorphism Network (GIN) with CNNs for high-dimensional feature extraction from SMILES, and rich pre-trained molecular semantic embeddings from a knowledge-based BERT (KB-BERT) model. LLMDTA [78] uses pre-trained biological LLMs (Mol2Vec for drugs and Evolutionary Scale Modeling-2 (ESM-2) for proteins) as feature extractors and employs a bilinear attention module. Table 1 provides a brief overview of such approaches that utilize language models for extracting features from biological entities.

**Table 1:** List of models utilized LLMs for feature encoding

**Model**	**Drug encoder**	**Protein encoder**	**Reference**
AMMVF-DTI	GAT	BERT	[100]
AttentionMGT-DTA	GAT	ESM-2	[56]
ConPLex	Morgan fingerprint	ProtBERT	[85]
DLM-DTI	ChemBERTa	ProtBERT	[79]
DrugLAMP	ChemBERTa-2	ESM-2	[93]
DTI-BERT	FP2 molecular fingerprint	ProtBERT	[74]
DTI-LM	ChemBERTa	ESM-2	[75]
DTIAM	BERMol	ESM-2	[104]
FusionDTA	One-hot encoding	ESM-1b	[84]
G-K BertDTA	KB-BERT	DenseSENet	[77]
LANTERN	ChemBERTa, MolFormer, MolT5	ProtT5, ProtBERT, ESM-3	[76]
LLMDTA	Mol2Vec	ESM-2	[78]
MMDG-DTI	SMILES-BERT	ProtBERT	[96]
MuFAl	Morgan fingerprint	ProtBERT	[40]
MultiKD-DTA	GINConv	ESM-2	[82]
Top-DTI	MolFormer	ProtT5	[94]

DLM-DTI [79] adopts a hint-based learning strategy with a compact student model that blends general and task-oriented knowledge. Due to including simple, fully connected layers, this approach performs poorly in cold-start scenarios. Potential strategies to handle the cold-start scenario could have been integrating a squeeze-and-excitation, capsule, and cross-attention network. MiRAGE-DTI [80] addresses the challenges existing DTI models face in effectively representing complex biological data, integrating diverse data sources, ensuring scalability, handling sparse and noisy datasets, and maintaining interpretability, along with class imbalance and data variability, by incorporating diverse drug and target similarity measures (structural, functional, and interaction-based) into a unified model.

GRA-DTA [81] employing a soft attention-based Bidirectional Gated Recurrent Unit (BiGRU) for protein features, GraphSAGE for drug graph representations, and an attention neural network. This approach showcased good results in the cold-start scenario when adequate unique drugs were present. MultiKD-DTA [82] addresses the limitations of GNNs susceptible to overfitting and CNNs/Transformers requiring fixed-length protein sequences leading to information loss by integrating a novel multiscale Wide and Deep CNN after GIN for enhanced drug feature exploration (both depth and breadth) and utilizing the ESM-2 pre-trained model with a BiLSTM network. To improve the interpretability of this approach, they applied t-distributed stochastic neighbor embedding for dimensionality reduction on the dataset.

#### The current frontier: multi-modal fusion, integration, and generation

Relying on the efficient feature learning capabilities of the attention mechanism, the current era is witnessing the amalgamation of multi-modal feature fusion, multi-level data stacking, and more PLM-based embedding generation while maintaining model interpretability.

Yang *et al.* emphasized dynamic learning by introducing a mutual learning mechanism using multi-headed attention and position-aware attention in their approach named ML-DTI [83], enabling bi-directional information flow between drug and target representations. It shows interpretability by mapping attention weights to protein sequences and compound molecules. Later, Yuan *et al.* proposed FusionDTA [84], which utilizes a multi-headed attention mechanism where the conventional rough pooling is replaced with global information aggregation and adopts a teacher-student model approach where learnable information is transferred from the teacher model to student model to reduce model complexity while maintaining performance, even in the cold-start scenario.

Singh *et al.* proposed ConPLex [85], which addressed that existing DTI methods suffer from ground-truth data scarcity and are highly dependent on physicochemical similarity-based modeling, which makes these models unable to distinguish true-positive binding compounds from physicochemically similar false positives (“decoys”). ConPLex handles these two limitations using two distinct modules: The “Con” module employs protein-anchored contrastive learning, which can distinguish positive compounds from decoys specifically, and the “PLex” module utilizes pre-trained protein language models (PLMs) to adopt knowledge from millions of protein sequences to handle the data scarcity issue.

Although attention mechanisms display superior binding prediction performance compared to other methods, binding prediction often relies on learned attention weights, lacking real-world biological insights. To mitigate this drawback, Hua *et al.* introduced MFR-DTA [86] that incorporates a BioMLP/CNN block along with a Mix-decoder block that relies on supervised learning with actual binding site information and learns by extracting element-wise and global features form biological sequences. In a similar approach, Ma *et al.* proposed a model named MSF-DTA [87] that gathers additional information from “neighboring” proteins in protein–protein interaction (PPI) and sequence similarity networks (SSNs) for more informed prediction.

To discuss the multi-modal approaches, Maroua *et al.* integrated tri-modality representations (molecular images, chemical sequences, and graph representations of drugs) to comprehensively capture structural, spatial, and functional aspects in their proposed approach called TriCvT-DTI [88]. Similarly, Debnath *et al.* proposed GramSeqDTA [89] that employs a Grammar Variational Autoencoder for drug feature extraction to learn semantic and syntactic rules and by fusing chemical perturbation (gene expression) information from the L1000 [90] project to incorporate functional features. HMSA-DTI [91] takes multiple drug and protein representations as input and employs a hierarchical multi-modal self-attention mechanism to fuse features deeply. MGSDTA [92] integrates both graph features and pre-trained sequence embeddings (from Mol2vec for drugs and ProtVec for targets) into a unified multi-modal framework with a weighted fusion module, suitable for handling cold-start problem. Luo *et al.* developed DrugLAMP [93] that leverages PLM and traditional feature extractors, coupled with novel multi-modal fusion modules (Pocket-Guided Co-Attention (PGCA) and Paired Multi-Modal Attention (PMMA)) and a contrastive pre-training module to align features across modalities and conditions. This model has outperformed previous state-of-the-art in cold-split settings and demonstrates high interpretability by visualization of attention maps.

Talo *et al.* proposed Top-DTI [94] to address the limitation of existing DTI prediction methods overlooking crucial topological components and structural data and facing challenges in cold-split scenarios for unseen drugs or targets by integrating Topological Data Analysis to extract topological features from protein contact maps and drug molecular images, while simultaneously employing LLMs to generate semantically rich embeddings from sequences. Another approach that explicitly handles the cold-split scenario is ColdDTA [95], which employs a data augmentation strategy that generates new drug-target pairs through subgraph removal and utilizing an attention-based feature fusion module to integrate drug and protein features better. Instead of a random split, the model performance is evaluated exclusively in cold-split settings. It provides model weights visualization from interpretability.

MMDG-DTI [96] addresses the limited generalization ability of existing deep learning DTI methods to unseen samples and unfamiliar domains, caused by their reliance on source-domain-restricted prior knowledge and susceptibility to redundant domain information by leveraging pre-trained LLMs for generalized textual features. They provide visualization of the high-response regions of protein–drug pairs for an interpretable verification of their method. Similar feature stacking-based approaches include DrugKANs [97] that integrates a dual-tower architecture with Kolmogorov–Arnold Network technology to enhance quality and interpretability, utilizing pre-trained models for initial representations, employing a lightweight attention mechanism and feature interaction to capture key features and mitigate overfitting. Developed by Shi *et al.*, SSCPA-DTI [98] is an interpretable approach that extracts both original and sub-structural features from drug and protein sequences using a multi-feature Information Mining Module (MIMM) and then interactively integrating these features and extracting interaction information using a Cross-public attention mechanism (CPA). MultiGranDTI [99] showcased an explainable multi-granularity representation framework that integrates atomic and sub-structural information for drugs via a hierarchical network and models multi-order sequence and spatial data for proteins. AMMVF-DTI [100] extracts interactive features from both node-level and graph-level embeddings, enabling more effective capture of local and global structural information.

Approaches that address the issue of interpretability include IMAEN [101], which employs a molecular augmentation mechanism and an interpretable stack convolutional encoding module. DrugAgent [102] integrates multiple specialized AI agents (ML, Knowledge Graph, Search) that leverage Chain-of-Thought and ReAct frameworks to provide transparent, human-interpretable reasoning for each prediction.

The unavailability of large-scale positive interaction data makes deep learning models poorly distinguish between binders and non-binders. Thus, Zhang *et al.*, in their proposed model named PLANET [103], proposed a multi-objective training approach that learns from non-binders collected from ChEMBL and performs tasks including affinity prediction, protein-ligand contact map prediction, and ligand distance matrix prediction. This approach allows contact map-based rescaling of energy contributions from interacting residue-atom pairs. Several other approaches adopted the multitask prediction architecture, namely DTIAM [104] employs self-supervised pre-training on vast amounts of unlabeled drug molecular graphs and protein sequences to learn comprehensive representations for accurate DTI, DTA, and MoA prediction. Shah *et al.* developed a novel multitask learning framework that simultaneously predicts DTB affinities and generates new target-aware drug variants using standard features called DeepDTAGen [105] that shows robustness in the DTA prediction through (i) drug selectivity, (ii) Quantitative Structure-Activity Relationships analysis, and (iii) cold-start tests.

## Popular benchmarks

This section discusses the most frequently used benchmarks for DTI and DTA tasks from the surveyed literature:

### BindingDB

BindingDB [20] contains experimental binding affinities between small molecules and protein targets and pharmacological annotations of the entities (e.g. ID, Structure etc.). The affinity records are sourced from popular biological database like ChEMBL [106], PDB [107], PubChem [108], and UniProt [109]. Even though the total no of records is very large, but researchers usually preprocess this dataset as per their requirement.

### BioSNAP

BioSNAP [110] was created by multiple researchers and is sourced from DrugBank [111] database. This dataset ensures a balance in the number of negative and positive samples.

### Caenorhabditis elegans

Curated by Liu *et al.* [112], *C.elegans* is a balanced DTI dataset. The positive instances are sampled from DrugBank [111], Matador [113], and STITCH [114] databases. Originally only contains positive samples, the highly credible negative samples are generated using an in silico screening method based on the assumption that similar compounds interact with the proteins similar to known proteins.

### Davis

As the survey suggests, Davis [115] is the most widely used dataset for benchmarking DTI and DTA models. Davis consists interaction information from the selectivity assay of the kinase protein family and corresponding inhibitors from their original experimental study. For DTA tasks, the affinity information between drugs and targets is given as K$_{d}$(dissociation constant) measurements.

### DrugBank

Curated by Zhao *et al.* [67], DrugBank is created by sourcing positive interactions from DrugBank (v5.1.5). Drugs which are inorganic compounds, very small molecule compounds [e.g. Iron (DB01592) and Zinc (DB01593)] or those of which the SMILES string cannot be recognized by RDKit [42] python package are then discarded. Further sampling is performed from the unlabeled drug–protein pairs to generate negative samples and obtain a balanced dataset with equal positive and negative samples.

### DTINet

Created by Luo et al [28], DTINet is a heterogeneous network. It contains four types of nodes—drugs, proteins, diseases and side effects, and the nodes are inter-connected by eight types of interactions, including drug-protein interactions. The nodes information are extracted form DrugBank(drugs), HPRD(proteins with UniProt IDs) [116], Comparative Toxicogenomics Database(disease) [117] and SIDER(side-efects) [118] databases.

### DUD-E

DUD-E [21] is a robust, high coverage dataset for structure-based virtual screening methods. It is a well-known benchmark consisting of 102 targets across 8 protein families. On average, each target has 224 actives and each active has 50 decoys. Decoys are chosen in such a way that they are physically similar but topologically dissimilar to the actives.

### Gold-standard

Created by Yamanishi *et al.* [10] by sourcing KEGG BRITE [119], BRENDA [120], SuperTarget [113], and DrugBank databases, this dataset contains high-quality positive interactions and is one of the earliest DTI benchmarks. It is consists of four sub-datasets: Enzymes, GPCRs, Ion Channels (ICs), and Nuclear Receptors (NRs).

### Human

Similar to *C.elegans* dataset, Human is a balanced DTI dataset is curated by Liu *et al.* [112], where the highly credible negative samples are generated based on the assumption that similar compounds interact with the proteins that are similar to known proteins.

### KIBA

Similar to Davis, KIBA [121] is one of the most widely used benchmarks for DTI and DTA tasks. KIBA contains the largest collection of binding informations for kinase family proteins and corresponding inhibitors sourced from widely used bioactivity databases like ChEMBL and STITCH. The binding affinity is measured using KIBA, which is a statistical combination of IC$_{50}$, K$_{d}$, and K$_{i}$ values.

### PDBBind

PDBbind [122] contains collection of experimentally validated DTB affinity samples from PDB, with available 3D structures of the drugs and targets. The dataset is distributed into three sub-datasets—general, refined and core. The general set contains all drug-target complexes from the PDB databses, the refined set contains refined, high-quality binding affinity measurements.

**Figure 2:**
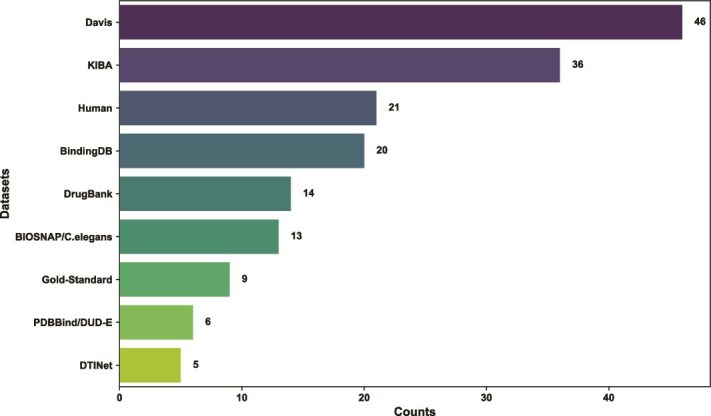
Frequency distribution of top ten most frequent datasets used in the surveyed methods.

Table 2 and Fig. 2 summarizes the statistics and the usage frequency of the aforementioned benchmarks, respectively.

**Table 2:** Statistics of most frequent benchmarks

**Dataset**	**Task**	**Drugs**	**Targets**	**Interactions**	**P/N Ratio**	**Drug length (max/avg)**	**Protein length (max/avg)**	**Label**	**Reference**
BindingDB	DTI,DTA	1 269 104	8810	$\sim $ 2 900 000	$P<N$	101/28	1485/239	0/1, pEC$_{50}$, pIC$_{50}$, pK$_{d}$, pK$_{i}$	[20]
BioSNAP^a^	DTI	4510	2181	27 464	1:1	-	-	0/1	[110]
*C.elegans*	DTI	1434	2504	7786	1:1	252/34	13100/530	0/1	[112]
Davis	DTI,DTA	68	442	30 056	$P \ll N$	103/64	2549/768	0/1, pK$_{d}$	[115]
DrugBank	DTI	6645	4254	35 022	1:1	250/55	14507/545	0/1	[67]
DTINet^a^	DTI	708	1512	1923	1:5	-	-	0/1	[28]
DUD-E^a^	DTI	22 886	102	1 167 186	$P \ll N$	-	-	0/1	[21]
Gold-standard^a^									
(Enzyme/IC/GPCR/NR)	DTI	445/210/223/54	664/204/95/26	2926/1476/635/90	Only +ve pairs	-	-	0/1	[10]
Human	DTI	2726	2001	6728	1:1	420/47	5038/623	0/1	[112]
KIBA	DTI,DTA	2111	229	118 254	1:4	590/50	4128/700	0/1, KIBA Score	[121]
PDBBind^a^^,^^b^	DTA	-	-	>27 000 (General), >7000 (Refined)	NA	-	-	pIC$_{50}$, pK$_{d}$, pK$_{i}$	[122]

^a^Drug and target length information not available.

^b^Concentrates on drug-target complexes; drug and target statistics unavailable.

## Evaluation metrics

The selection of appropriate evaluation metrics is of utmost importance for a thorough comparative analysis of the performances of different computational approaches for DTI prediction. Here, we discuss some of the most frequently used evaluation metrics in DTA and DTI prediction tasks.

### Evaluation metrics for DTI prediction

In DTI predictions, the goal is to classify the likelihood of interaction between a drug-target pair. An interaction is positive if a drug-target pair is likely to interact, otherwise negative. In classification tasks, performance of a model is usually represented using the following components: **True positive (TP), True Negative (TN), False Positive (FP),** and **False Negative (FN)**.

**TP** denotes the number of correctly predicted positive labels.

**TN** denotes the number of correctly predicted negative labels.

**FP** denotes the number of instances where the actual label is negative but the model predicts it as positive.

**FN** denotes the number of instances where the actual label is positive but the model predicts it as negative.

Evaluation metrics for DTI prediction are constructed combining the aforementioned components:

#### Accuracy

Accuracy is defined as the fraction of correctly predicted labels, calculated as the sum of correctly predicted positive and negative instances over total number of predictions. The fraction is often multiplied by 100 to transform into a percentage.


(1) \begin{align*}& \text{Accuracy} = \frac{TP + TN}{TP + TN + FP + FN}\end{align*}

#### Precision

Precision, aka Positive Predictive Value is the fraction of correctly predicted positive instances, calculated as the number of correctly predicted positive labels over all the predicted positive labels.


(2) \begin{align*}& \text{Precision} = \frac{TP}{TP + FP}\end{align*}

#### Recall

Recall, aka Sensitivity or True Positive Rate (TPR) is the fraction of actual positives correctly identified, calculated as the number of correctly predicted positive labels over all the actual positive labels.


(3) \begin{align*}& \text{Recall} = \frac{TP}{TP + FN}\end{align*}

sectionF1 Score F1 Score is defined as the harmonic mean of precision and recall, balancing both to measure the accuracy of a model handling false positive and false negatives, especially useful for imbalanced datasets.


(4) \begin{align*}& F_{1} = 2 \times \frac{\text{Precision} \times \text{Recall}}{\text{Precision} + \text{Recall}}\end{align*}

#### AUPR

Area Under the Precision-Recall Curve (AUPR) measures the trade-off between precision and recall, especially useful in imbalanced datasets where positive class is rare.


(5) \begin{align*}& \text{AUPR} = \int_{0}^{1} \text{Precision}(r) \cdot d\text{Recall}(r)\end{align*}

#### Area under the receiver operating characteristic curve

Area under the receiver operating characteristic curve (AUROC) evaluates the ability of a model to distinguish between classes by calculating the area under the ROC curve plotting TPR against False Positive Rate (FPR) across different thresholds.


(6) \begin{align*}& \text{AUROC} = \int_{-\infty}^{\infty} \text{TPR}(t) \cdot d\text{FPR}(t)\end{align*}

### Evaluation metrics for DTA prediction

In DTA prediction, the goal is predict a continuous label representing the degree of interaction between a drug-target pair. These labels are usually interaction coefficients like $ K_{d} $, $ K_{i} $, $ IC_{50} $, or $ EC_{50} $.

$ K_{d} $ (dissociation constant) is the equilibrium concentration at which half of the ligand-target complex dissociates, indicating binding affinity. Lower the value of $ K_{d} $, higher the affinity between ligand-target complex. Similarly, $ K_{i} $ (inhibition constant) represents the binding affinity of an inhibitor to its target, independent of the substrate concentration. Lower $ K_{i} $ indicates stronger affinity.

$ IC_{50} $ (inhibitory concentration 50%) signifies the concentration of an inhibitor required to reduce a biological activity (e.g. enzyme function) by 50%. Similarly, $ EC_{50} $ (effective concentration 50%) denotes the concentration of a drug that produces 50% of its maximum effect.

Evaluation metrics for DTA prediction predicted are as follows:

#### Concordance index

Concordance index (CI) measures how well a model ranks predicted labels relative to the actual ones by evaluating concordant pairs—instances where the model predicts higher value for an actual high value label. A concordant pairing occurs when the predicted ranking is well aligned with the actual ranking, while a pair is called discordant when the ranking is incorrect. A CI value of 1.0 indicates perfect ranking while a vlue of 0.5 represents random performance.


(7) \begin{align*}& CI = \frac{1}{Z} \sum_{y_{i}> y_{j}} h\left(\hat{y}_{i} - \hat{y}_{j}\right)\end{align*}

where, $y_{i}$, $ \hat{y}_{i} $, $Z$, and $h$ are actual labels, predicted labels, total number of comparable pairs, and a step function, respectively.

#### Mean squared error

Mean squared error (MSE) measures the average squared differences between actual and predicted labels.


(8) \begin{align*}& MSE = \frac{1}{n} \sum_{i=1}^{n} \left(y_{i} - \hat{y}_{i}\right)^{2}\end{align*}

#### Pearson correlation coefficient

Pearson correlation coefficient (PCC) measures linear correlation between predicted and actual labels, with +1 being a perfect negative correlation and +1 being a perfect positive correlation. It is one of the most widely used metrics used to capture the linear trend between the predicted and ground truth values.


(9) \begin{align*}& r = \frac{\sum_{i=1}^{n} (y_{i} - \bar{y}) (\hat{y}_{i} - \bar{\hat{y}})} {\sqrt{\sum_{i=1}^{n} (y_{i} - \bar{y})^{2}} \sqrt{\sum_{i=1}^{n} (\hat{y}_{i} - \bar{\hat{y}})^{2}}}\end{align*}

#### $r_{m}^{2}$ Score

First introduced in [30], $r_{m}^{2}$ score penalizes overfitting, assuring the accuracy and robustness of the model.


(10) \begin{align*}& r_{m}^{2} = R^{2} \times \left(1 - \sqrt{|R^{2} - R_{0}^{2}|} \right)\end{align*}

where, $R_{0}^{2}$ is a variant of $R^{2}$, Coefficient of Determination, computed without an intercept in the regression. $R^{2}$ can be defined as:


(11) \begin{align*}& R^{2} = 1 - \frac{\sum_{i=1}^{n} (y_{i} - \hat{y}_{i})^{2}}{\sum_{i=1}^{n} (y_{i} - \bar{y})^{2}}\end{align*}

**Figure 3:**
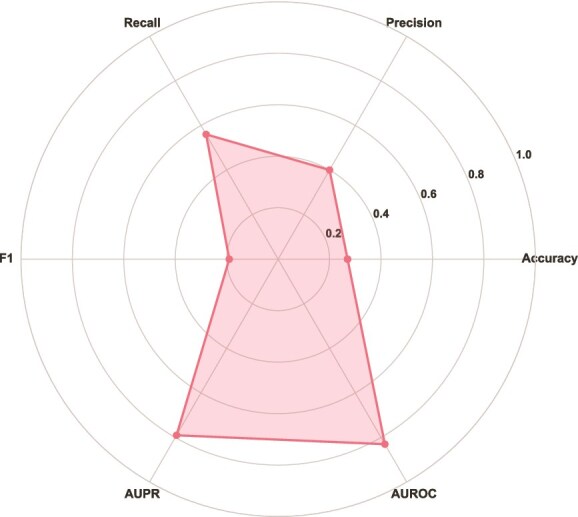
Frequently used metrics across DTI prediction methods: (a) Accuracy, (b) Precision, (c) Recall, (d) F1, (e) AUPR, and (f) AUROC.

**Figure 4:**
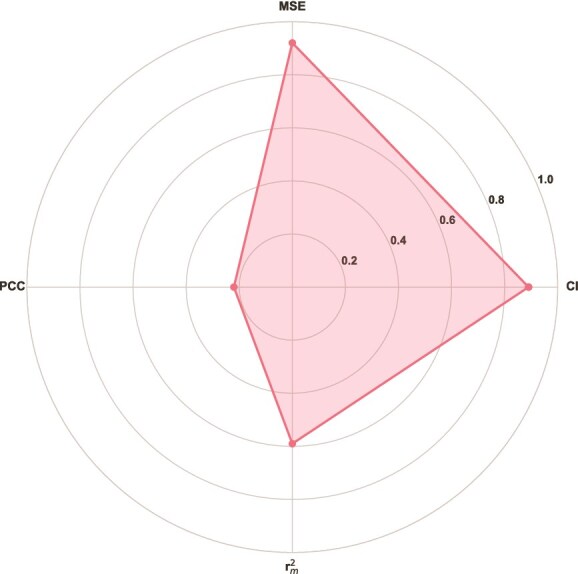
Frequently used metrics across DTA prediction methods: (a) CI, (b) MSE, (c) PCC, and (d) r$^{2}_{m}$.

Figures 3 and 4 display the distribution of the most frequently used metrics in surveyed DTI and DTA prediction methods, respectively.

## Evaluation methodology

### Model selection

**Figure 5:**
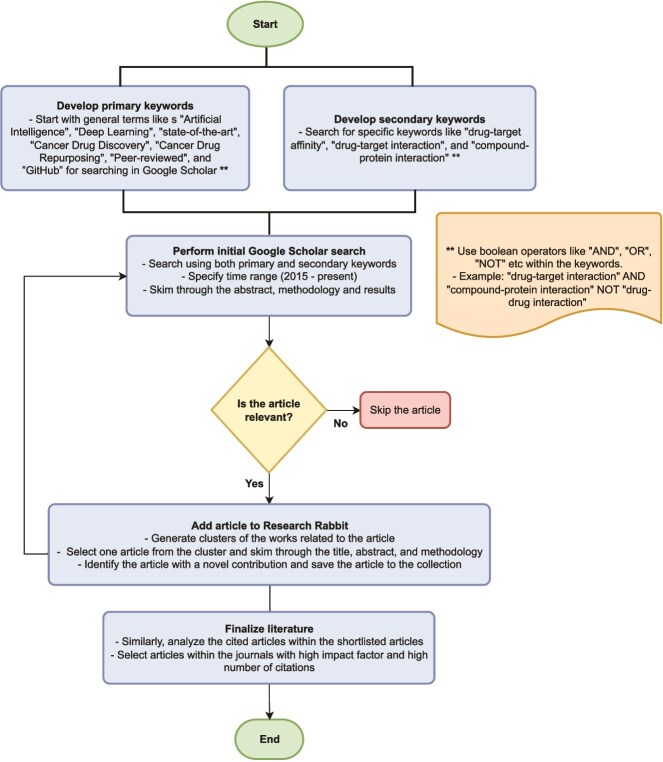
Methodology of article selection procedure for DTB prediction and related topics.

Conducting a comprehensive literature review is essential for identifying state-of-the-art evaluation techniques. This section outlines the methodology for selecting relevant works, primarily leveraging Google Scholar. Search queries were refined with keywords such as “AI,” “Deep Learning,” “state-of-the-art,” “Cancer Drug Discovery,” “Cancer Drug Repurposing,” “Peer-reviewed,” and “GitHub.” The search results were initially screened by evaluating the abstract, methodology, and results sections to determine relevance. Selected articles were then organized using Research Rabbit, a citation-based research mapping tool. Research Rabbit was further utilized to explore related works and trace relevant citations. Priority was given to studies offering comparative analyses of existing techniques. Additionally, the availability of open-source code was considered to facilitate ease of evaluation. The model selection procedure is discussed in detail in Fig. 5. Tables 3 and 4 provide brief descriptions about the DTI and DTA models selected for evaluation in this work, respectively.

**Table 3:** Methodological Description of Deep Learning Architectures for DTI Prediction

**Model**	**Description**	**Reference**
AMMVF-DTI	Uses GAT and BERT to capture local and global structural features	[100]
FragXsiteDTI	Leverages transformer-associated learnable latent array to translate between molecular fragment representations and protein pocket information	[73]
GraphsformerCPI	Integrates semantic and spatial structural features of compounds and proteins using structure-enhanced self-attention	[68]
IHDFN-DTI	Utilizes hybrid deep feature extraction for proteins that captures multi-level information via dynamic encoding	[65]
IMAEN	Employs molecular augmentation mechanism along with interpretable stack convolutional encoding module	[101]
MHSADTI	Leverages GAT for drugs to reduce the affect of noisy connections within the nodes of drug graph structure	[47]
MSF-DTA	Gathers node-level information from “neighboring” proteins in PPI and SSN networks	[48]
MultiGranDTI	Introduces multi-granularity representation framework that integrates atomic and sub-structural information for drugs via a hierarchical network	[99]
SSCPA-DTI	Uses MIMM and CPA for feature extraction and integration	[98]
TransformerCPI	Leverages self-attention mechanism for dynamic feature extraction	[61]

**Table 4:** Methodological description of deep learning architectures for DTA prediction

**Model**	**Description**	**Reference**
3DProt-DTA	Incorporates AlphaFold structure predictions in conjunction with graph representations of proteins	[44]
Affinity2Vec	Formulates the affinity prediction task as a graph problem using a weighted heterogeneous graph	[34]
DeepDTA	Uses CNNs to model protein sequences and compound 1D representations	[30]
DeepNC	Proposes multiple GNN algorithms (GENConv, GCNConv, and HypergraphConv) to learn features from drug structures	[43]
FusionDTA	Performs global feature aggregation utilizing a multi-head linear attention mechanism along with knowledge distillation	[84]
GRA-DTA	Employs BiGRU for protein features and GraphSAGE for drug graph representations	[81]
GraphDTA	Represents drugs and proteins as graphs and uses GNNs	[41]
GS-DTA	Uses hybrid GATv2-GCN for hierarchical drug feature extraction and CNN, BiLSTM, and Transformer for multi-level (local, contextual, global) protein feature extraction	[53]
ML-DTI	Proposes a mutual learning approach simultaneously from drug and target encoders using multi-headed and position-aware attention	[83]
MultiGranDTI	Introduces multi-granularity representation framework that integrates atomic and sub-structural information for drugs via a hierarchical network	[99]

### Evaluation

In this section, we discussed about the evaluation outcomes for each of the selected models. The models were evaluated using key performance metrics—AUROC for DTI models and MSE and CI for DTA models. The performance variation across different models can be attributed to the diversity of their underlying architecture and modes of feature representations. Tables 5–7 showcases the performance of the models when evaluated on Human dataset for DTI models and Davis and KIBA dataset for DTA models, respectively.

**Table 5:** Evaluation of DTI models on human dataset

Model	AUROC
AMMVF-DTI	0.986
FragXsiteDTI	0.991
GraphsformerCPI	0.990 $\pm $ 0.002
IHDFN-DTI	0.986 $\pm $ 0.001
IMAEN	0.954
MHSADTI	0.988 $\pm $ 0.001
MSF-DTA	0.982
MultiGranDTI	0.978
SSCPA-DTI	0.992
TransformerCPI	0.973 $\pm $ 0.002

**Table 6:** Evaluation of DTA models on Davis dataset

Model	MSE	CI
3DProt-DTA	0.184	0.917
Affinity2Vec	0.248	0.889
DeepDTA	0.245	0.881
DeepNC	0.273	0.876
FusionDTA	0.198	0.912
GRA-DTA	0.225	0.897
GraphDTA	0.267	0.878
GS-DTA	0.213	0.903
ML-DTI	0.211	0.887
MultiGranDTI	0.237	0.907

**Table 7:** Evaluation of DTA models on KIBA dataset

Model	MSE	CI
3DProt-DTA	0.141	0.891
Affinity2Vec	0.253	0.846
DeepDTA	0.191	0.858
DeepNC	0.162	0.887
FusionDTA	0.143	0.898
GRA-DTA	0.142	0.891
GraphDTA	0.159	0.877
GS-DTA	0.124	0.895
ML-DTI	0.202	0.864
MultiGranDTI	0.197	0.889

Based on the evaluation results among DTI approaches on the Human dataset, we can observe that models focusing on multi-level and more sub-structure-specific methodologies show the best results, while models focusing on global-scale structural features only showcase sub-optimal performance. Most best-performing models integrate attention mechanisms and visualization capabilities that provide biological insights into binding mechanisms and critical interaction sites: FragXsiteDTI (AUROC 0.991) uses transformer architecture focusing on drug fragments and protein pockets; SSCPA-DTI (AUROC 0.992) extracts both original and sub-structural features through specialized mining modules; GraphsformerCPI (AUROC 0.990) treats molecules as structured sequences with dual-attention mechanisms. Meanwhile, IMAEN, which is designed for interpretability, comparatively shows lower performance (AUROC 0.954).

Evaluation of DTA methods on both the Davis and KIBA datasets shows that models using attention-based methods outperformed graph convolution and classical sequence-based approaches. 3DProtDTA (best CI 0.891 in Davis) uses AlphaFold predictions, FusionDTA (best CI 0.898 in KIBA) incorporates global feature aggregation using multi-headed attention, GS-DTA (CI 0.903, 0.895 in Davis and KIBA, respectively) uses GAT along with Transformer; are the best-performing models. While models including DeepNC (CI 0.876 in Davis) utilize graph convolution methods, GraphDTA (CI 0.878 in KIBA) uses trivial graph representation, and DeepDTA (CI 0.858 in KIBA) uses CNNs to extract features from 1D sequences; shows sub-optimal performance.

## Case studies: predictive modeling for drug–target binding in cancer

Case studies have been performed to predict potential compound-target relationships. We used a dataset of lead-like compounds from the GDB-17 database, with molecular weights (MWs) ranging from 100 to 350 and clogP values between 1 and 3. This dataset, consisting of $\sim $8 million compounds [123], was curated to exclude molecules with small rings containing 3 to 4 atoms. For the protein targets, we selected few of the most researched key proteins involved in cancer pathways from the GDSC database, as listed below: EGFR (Pathway: EGFR signaling, UniProt: P00533), Tyrosine-protein kinase ABL1 (Pathway: ABL signaling, UniProt: P00519), Serine/threonine-protein kinase mTOR (Pathway: P13K/MTOR signaling, UniProt: P42345), Cellular tumor antigen p53 (Pathway: P53, UniProt: P04637), Mitogen-activated protein kinase kinase kinase 1 (MAP3K1) (Pathway: ERK/MAPK signaling, UniProt: Q13233). Table 8 summarizes all target proteins.

**Table 8:** Target proteins selected for case studies from GDSC database with corresponding gene, UniProt ID and pathway information

**Protein**	**Gene**	**Uniprot ID**	**Pathway**
Endothelial Growth Factor Receptor	EGFR	UniProt: P00533	EGFR signaling
Tyrosine-protein kinase ABL1	ABL1	UniProt: P00519	ABL signaling
Serine/threonine-protein kinase mTOR	MTOR	UniProt: P42345	P13k/MTOR signaling
Cellular tumor antigen p53	TP53	UniProt: P04637	P53 pathway
Mitogen-activated protein kinase kinase kinase 1	MAP3K1	UniProt: Q13233	ERK/MAPK signaling

EGFR is a tyrosine kinase receptor protein crucial in activating several signaling cascades to convert extracellular cues into designated cellular responses [124]. It regulates cell growth and survival and is frequently targeted in cancer therapy. Often, it is associated with lung cancer [125] and Neonatal nephrocutaneous inflammatory syndrome [126] development.

ABL1 is a non-receptor tyrosine kinase associated with key cell growth and survival processes, including cytoskeletal remodeling in response to extracellular stimuli, cell motility and adhesion, DNA damage response, and apoptosis [127]. It utilizes Mg2+ as its cofactor to operate. Chromosomal aberrations in ABL1 lead to fusion with the BCR gene, forming the oncogenic BCR-ABL1 complex, resulting in chronic myeloid leukemia [128]. ABL1 is also associated with acute lymphoblastic leukemia [129] and Congenital heart defects and skeletal malformations syndrome [130].

Associated with direct or indirect phosphorylation of over 800 proteins, mTOR is a Serine/threonine-protein kinase that is central to PI3K/AKT/mTOR pathway, playing a pivotal role in cellular growth and metabolism, response to hormones, growth factors, and other external signals [131]. It is part of two distinct signaling complexes, mTORC1 and mTORC2 [132]. It is associated with Smith–Kingsmore syndrome [133].

Upon binding with its target DNA sequences, p53 acts as a multifunctional transcription factor that regulates cell cycle arrest, DNA repair, and apoptosis, thereby acting as a tumor suppressor [134]. Uses Zn2+ as a cofactor. Mutations in p53 result in several types of cancers, including Esophageal cancer [135] and Li–Fraumeni syndrome [136].

MAP3K1 is an upstream regulator of the Extracellular signal-Regulated Kinase/Mitogen-Activated Protein Kinase (ERK/MAPK) signaling pathway. It activates the ERK and c-Jun N-Terminal Kinase (JNk) pathways by phosphorylating MAP2K1 and MAP2K4 [137]. It undergoes frequent alterations in various cancers, including breast and prostate cancer [138, 139].

Essentially the case studies can be divided into two categories: (i) case study for DTI prediction, and (ii) case study for DTA prediction.

### Case study-I: DTI prediction in cancer

We chose FragXsiteDTI as the inference model to run a case study for DTI predictions, as it shows exceptional AUROC values when evaluated with the Human dataset. Moreover, FragXsiteDTI utilizes a state-of-the-art transformer-based approach for analyzing fine-grained interaction between compound fragments and protein binding pocket, avoiding the consideration of redundant complete structures of the compound and protein. DTI prediction aims to calculate the probability of binding between a drug and a target pair. The probability can lie between 0 and 1. We chose a threshold value of 0.5, below which we can nullify the interaction. Against all five proteins selected as targets, we iteratively test the GDB-17 compounds.

### Case study-II: DTA prediction in cancer

FusionDTA is used for inference involving DTA predictions on GDB-17 compounds. We chose FusionDTA as it has fewer failure rates than DTA models working with 3D structural protein data, which sometimes tend to be sensitive to the quality, conformational state, and structural flexibility of the 3D protein structural data. Moreover, FusionDTA was one of the early attempts where a language model (ESM-1b) was incorporated in a DTB prediction architecture. In addition, FusionDTA pioneered the handling of the cold-start problem. In summary, the multi-dimensional functionality of the FusionDTA approach made us choose FusionDTA as our go-to model for the large-scale downstream analysis.

### In silico downstream analysis—evaluate drug-likeness

The predicted compounds require rigorous experimental validation to substantiate their efficacy on the selected targets. Experimental validations of these extensive predictions can be exponentially time-consuming and costly. However, we can mitigate the risk of nullifying the predictions by conducting in silico downstream analysis to gain estimates on their performance within a living system. Therefore, we performed further downstream analysis on the predicted compounds to evaluate their drug-likeness. In this analysis, we tested the compounds against some criteria crucial to drug development.

We applied Lipinski’s Rule of Five [140] and the Veber test [141]. According to Lipinski’s Rule of Five, a compound is considered drug-like if it satisfies the following conditions:

-
$MW$ $\leq 500$ Da
- LogP (Lipophilicity) $\leq 5$
- Hydrogen Bond Donors $\leq 5$
- Hydrogen Bond Acceptors (HBAs) $\leq 10$

Veber’s Rule checks the molecular flexibility and permeability of a compound, which aids in oral bioavailability. It states that a drug may have good oral bioavailability if it meets the following criteria:

- Topological Polar Surface Area (TPSA) $\leq 140$ Å$^{2}$
- Number of Rotatable Bonds (RBs) $\leq 10$

We also assessed basic criteria that define the ADMET (Absorption, Distribution, Metabolism, Excretion, and Toxicity) [142, 143] profile of a drug-like compound, specifically solubility, permeability, hERG risk [144], and the AMES test [145]. Another critical metric is the blood–brain barrier (BBB) permeability, which is defined by the ability of a drug to traverse the protective barrier that restricts the entry of harmful substances from the bloodstream into the brain while permitting the passage of essential nutrients. However, this criterion is only relevant for drugs of the central nervous system [146]. Various physicochemical properties contribute to defining these criteria, with their acceptable values outlined as follows:

- Solubility: $\log P \leq 5$
- Permeability: RBs $< 5$, TPSA $< 70$ Å$^{2}$
- hERG Risk: $\log P> 4$
- AMES Test: $(MW)> 350$, HBAs $> 5$
- BBB Permeability: $2 < \log P < 4$

Absorption of a drug through the gastrointestinal (GI) lining plays a crucial role in its distribution and, consequently, its effect on the designated target [147]. Certain criteria must be met for GI absorption, which are as follows:

- TPSA $\leq 140$ Å$^{2}$
- Number of RBs $\leq 10$

Synthetic accessibility [148, 149] defines the ease of synthesis of a compound, ranging from 1 (easily synthesizable) to 10 (difficult to synthesize). It is a crucial aspect of drug discovery, particularly in the synthesis of *de novo* drugs.

**Figure 6:**
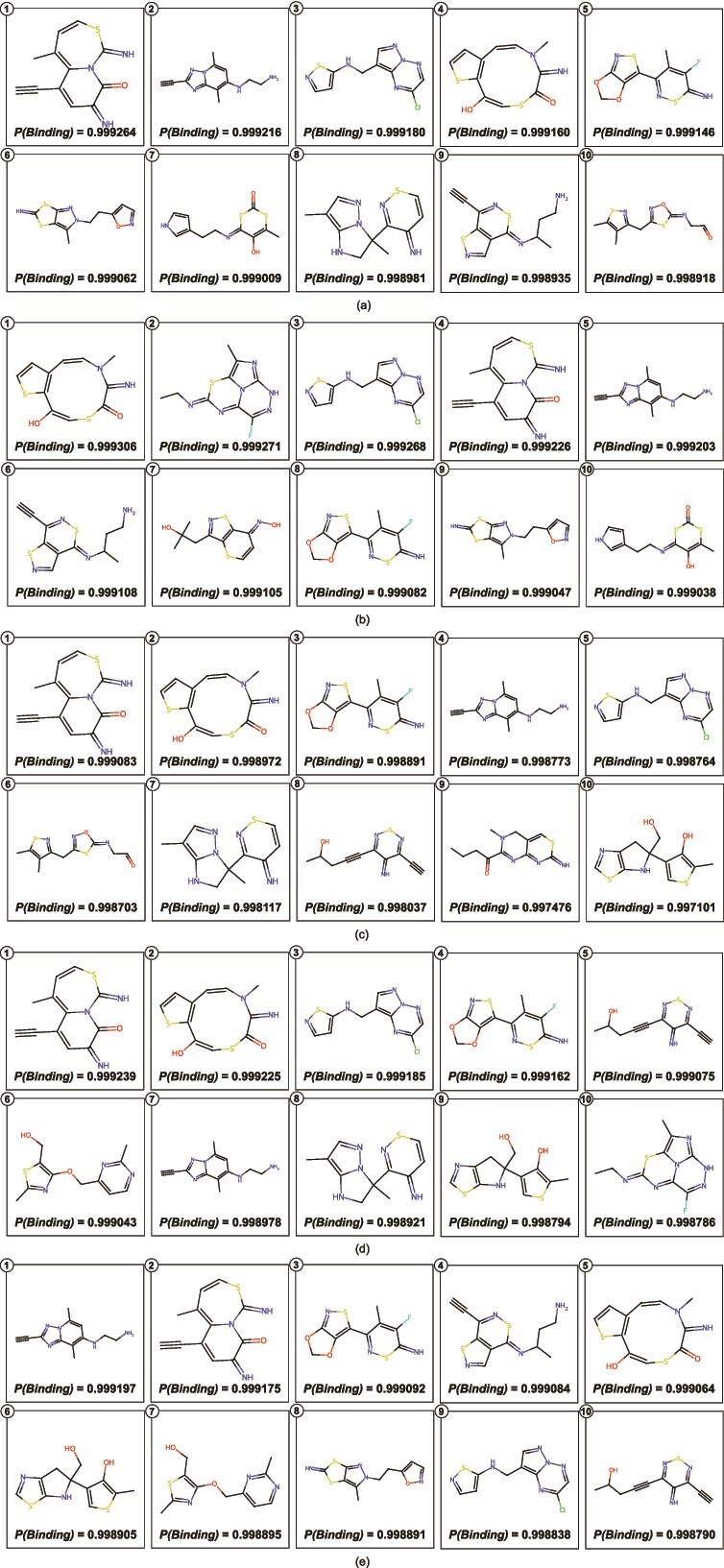
Top ten predicted compounds with highest binding probabilities against (a) Endothelial Growth Factor Receptor, (b) Tyrosine-protein kinase ABL1, (c) Serine/threonine-protein kinase mTOR, (d) Cellular tumor antigen p53, and (e) MAP3K1.

**Figure 7:**
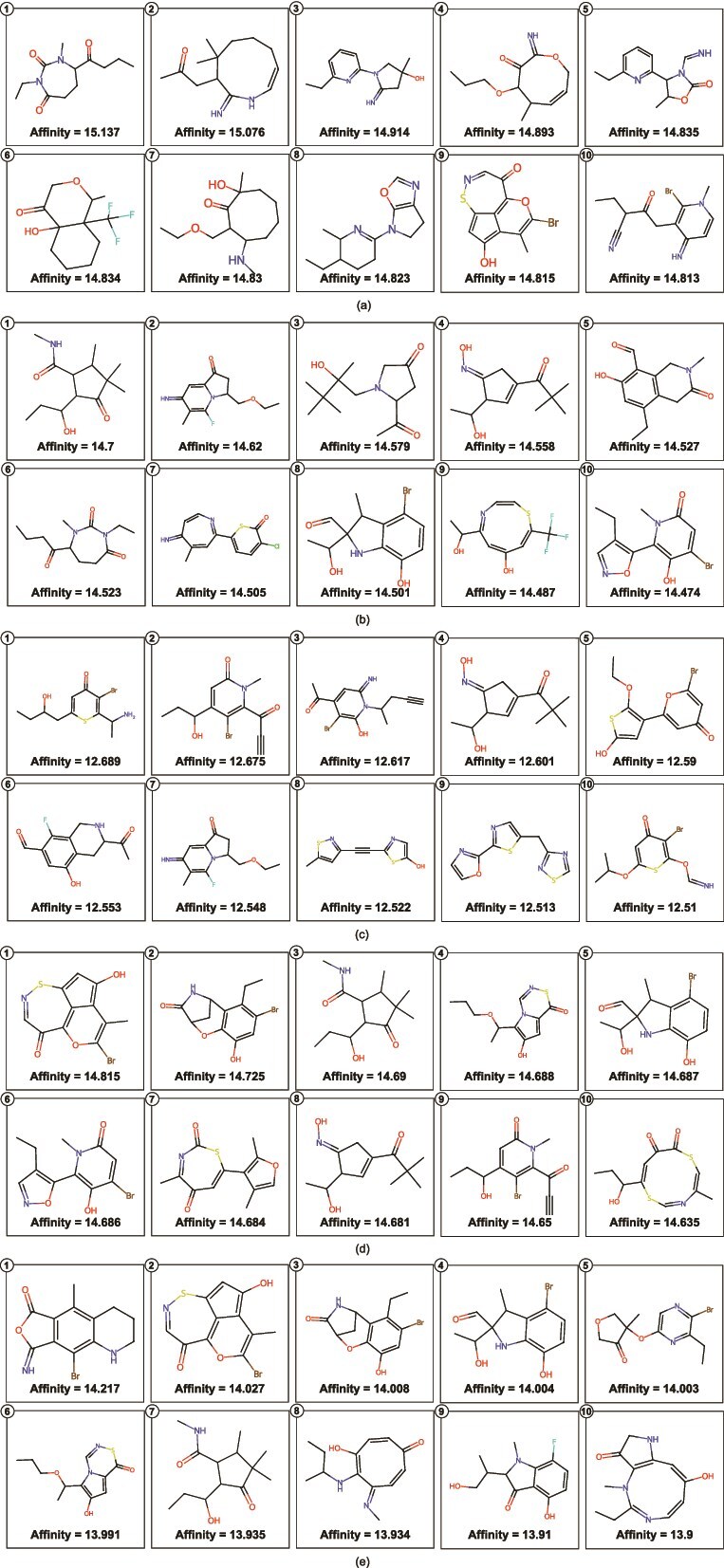
Top ten predicted compounds with highest KIBA scores against (a) Endothelial Growth Factor Receptor, (b) Tyrosine-protein kinase ABL1, (c) Serine/threonine-protein kinase mTOR, (d) Cellular tumor antigen p53, and (e) MAP3K1.

The in silico analysis emphasizes the significance of drug-likeness filters, as the filtered compounds meet the physicochemical requirements essential for being lead compounds. The analysis results identify those compounds with the potential to pass experimental validation. We showed the top ten compounds predicted to have the highest probabilities to bind to each target protein in Fig. 6, all of which meet the drug-likeness criteria mentioned above. Importantly, we also mention the predicted probabilities of binding along with the figures for comprehension. Similarly, Fig. 7 presents the top ten predicted compounds with the highest binding affinity scores for selected target proteins. As for the DTA case study, the predictions were made using the FusionDTA model pre-trained on the KIBA dataset; the *Affinity* values represent KIBA scores for compound-target pairs.

Case studies suggest that most of the compounds predicted with high binding probabilities in the DTI case study also showcase good binding affinities for the DTA case study, which emphasizes the mutual accountability and coherence of the case studies. Notably, most top-ranked compounds in each case study exhibit aromatic, cyclic, and heterocyclic functional groups. Literature suggests these structural motifs are prevalent in known protein kinase inhibitors and other cancer-related therapeutic agents. These findings underscore the importance of the predicted compounds as potential lead candidates for targeting kinases in cancer therapies, with room for further modifications. This analysis bridges the gap between computational prediction and practical drug development.

Approaches like multi-omics integration for personalized target selection and incorporating tumor heterogeneity data to predict context-specific interactions can provide potential means of addressing critical oncology-specific challenges through DTB models. However, current approaches struggle with the dynamic evolution nature of cancers and lack sufficient training data for rare mutations. Off-target toxicity prediction remains inadequate due to an incomplete understanding of cancer-specific protein interactions and resistance mechanisms.

## Challenges and limitations

Classical and reverse pharmacology approaches have limitations in identifying new molecular entities (NMEs). The process is time-consuming, expensive, and often has high failure rates. Deep-learning-based drug discovery approaches hold immense potential in developing NMEs for various diseases. However, some challenges and limitations must be addressed and bypassed for effective implementation of the approaches.

One major challenge is the quality and quantity of the available data resources. Deep learning models thrive on large volumes of high-quality data for training. Researchers must ensure that the data for training deep learning models are precise, representative, and related to specific research objectives. Addressing data limitations, such as biases or incomplete datasets, is prioritized to avoid skewed results and unreliable conclusions. For instance, two of the most used benchmark datasets in DTB prediction models are Davis and KIBA—which we also used for our evaluation task. Both of these datasets represent kinase–inhibitory compounds in their drug set. Models trained on these two datasets thus only capture drug features effective against kinase and similar proteins. Even though, as per the studies, kinase proteins play a crucial role in various cancer progressions, targeting only those proteins creates a void in research focusing on related proteins playing a crucial role in the overall cancer microenvironment. Curating such a dataset, which includes other important kinase proteins, is highly important to avoid bias toward developing compounds effective against kinase-proteins only.

Considering effective representation strategies for drugs and proteins is also a crucial aspect of constructing a model. From the evaluation outcomes above, it is evident that structure-based approaches perform better than the models considering sequence information only. The reason can be the definite topological skeleton information carved in the structural representations of the biological entities, which aids the models in extracting meaningful and natural biological features from those representations. Upon administration, drugs enter the human system in their complete structural form (3D), which is then transformed into subsequent simpler forms through absorption, digestion, and metabolism. Thus, considering the complex structural form as the representation’s starting point is the most meaningful approach to mimic the natural compound activity inside the human system. The same goes for the target proteins. Proteins take their most stable quaternary form inside the human system. Thus, considering that particular form as the starting point and extracting biologically relevant structural features from those makes the utmost sense. Although graph-based approaches can model complex representations of biological entities from their primary representations, experimentally validated complex structures are more trustworthy and meaningful. Resources like AlphaFold are gaining popularity with their highly accurate predicted 3D protein structures, but such a resource for compounds is still unavailable.

Experimental validation of the predictions adds more weight to the outcomes, as the final goal is to design a real-life compound to be administered in the human system. The cycle starts with lab-scale testing of the compound, followed by a mouse model, and finally, clinical trials on humans. The absorption, digestion, metabolism, and excretion profile must be monitored upon administering a compound in a human system. The process is standardized and proven effective in analyzing a compound’s efficacy and side effects before proceeding with its large-scale manufacturing. Nevertheless, starting from the lab-scale experiments and industry-scale manufacturing, the process demands a lot of time and economic resources, often with high failure rates. On top of that, there are frequent regulatory alterations and ethical concerns. Thus, instead of de novo compound design, drug repurposing is gaining much importance, which talks about using existing drugs that have proven effective for one disease and can be used for another.

In summary, deep learning-driven DTB prediction presents exciting opportunities, but the aforementioned concerns must be addressed. Deep learning approaches provide possibilities for revolutionizing the field by speeding up the identification of DTBs. However, the challenges and limitations mentioned above emphasize the importance of interdisciplinary collaboration, better data curation, innovative yet practical representations, and ethical and scientific considerations. Overcoming this obstacle will lead to the successful integration of deep learning in DTB prediction and ultimately fuel innovation across the computational drug discovery domain.

## Discussion and future scope

The AI-driven drug discovery market is growing daily and is projected to grow significantly in the coming years. Advancements in AI technologies, such as machine learning, deep learning, and natural language processing, have developed sophisticated algorithms capable of analyzing complex biological data and predicting DTBs. Deep learning models actively analyze large compound libraries and databases to identify potential compounds against specific targets and diseases with higher accuracy and efficacy than traditional methods. Nevertheless, each methodology class evaluated for DTB prediction has advantages and disadvantages.

Sequence-based methods are good when the primary sequence information for compounds and targets is available, but they often lack generalizability and struggle with capturing high-dimensional molecular interactions. Graph-based methods can effectively analyze structural dependencies in molecular graphs, but they are computationally heavy and often depend on the availability of high-quality structural data. Attention-based methods can model long-range dependencies, and utilization of multi-headed attention enables better feature aggregation but often requires large datasets for optimal performance and is sensitive to dataset biases and inconsistencies. Hybrid models integrate multiple representations and embeddings to enhance affinity prediction but often result in complex model architectures. Studies also suggest that models that are pre-trained on large amount of relevant molecular/biological data are more suitable for small-sample learning.

The literature from both the pre-deep and post-deep learning eras shows that integrating additional information other than drug and protein improves the DTB prediction efficiency. Supplementary information, including PPI, drug-drug interaction, drug-disease relationship, gene-disease relationship, side-effect information, and chemical perturbation information, play crucial roles in DTB prediction by working as an additional modality.

Targeting specific proteins in human proteomes is a conventional practice in targeted cancer therapy. The pre-deep learning era was all about trial and error testing and statistical analysis-driven approaches for DTB predictions on selected proteins, which is now directed toward a more precise approach leveraging the data-driven decisions post-deep learning era. The human genome contains an estimated 6000–8000 potential drug targets, but only a tiny fraction have been exploited for drug development. This vast untapped potential highlights the need for continued research and development in DTB prediction. The availability of experimentally validated and high-confidence predicted structural data sources will play a crucial role here.

To effectively address critical data quality issues including label bias and class imbalance in model training and generalization, researchers must systematically adopt comprehensive intervention strategies. This specifically requires deploying advanced sampling techniques like SMOTE and cost-sensitive learning, establishing robust multi-source annotation protocols with uncertainty quantification, and implementing sophisticated ensemble frameworks with adaptive loss functions. These strategic approaches will ensure highly reliable, generalizable DTB prediction models that can effectively overcome fundamental dataset limitations completely.

The cold-start problem arises when models perform poorly when tested on unseen data, which reflects their performance in real-world scenarios. Various approaches have been adopted to improve model performance in cold-start scenarios. One such approach is enabling domain adaptation through adversarial training, which addresses the distribution shifts between training and test scenarios. An alternative strategy involves capturing robust, generalizable features using methods like feature fusion and representation learning. A further methodology discusses generating diverse drug-target pairs by systematically removing drug sub-graphs to enhance topological generation. Another complementary approach is to adopt a meta-learning framework that enables adaptation to novel scenarios by learning optimal parameter initialization strategies from historical tasks. In more recent approaches, pre-trained molecular representations sourced from language models are used to get the structural priors of biological entities that improve zero-shot prediction capabilities. However, challenges remain in making biologically and chemically feasible predictions, which is an active area of research and requires major innovation.

Most of these DTB models usually get integrated in industry-scale drug discovery piplines as early stage filters and are often combined with ADMET prediction models for pharmacokinetic filtering. Integration occurs through APIs or embedded modules that output confidence scores and binding predictions. However, their real-world impact remains limited—most models show poor performance on novel targets due to training bias toward well-studied proteins.

The future of DTB prediction lies in leveraging advancements in computational power, data analysis, and AI to develop more sophisticated and accurate models. With an increasing number of advanced DTB models, concerns regarding the ’black box’ limitations of these models are increasingly prominent. Several approaches incorporate interpretability to their models using methods namely attention weight visualization, attention focusing and mapping attention weights back to protein sequences and compound molecules. These models adopt the approach of domain-specific knowledge integration. However, these models are computationally demanding and often face the challenge of knowledge graph bias. These limitations must be addressed in future developments.

As regulatory agencies, such as the FDA, continue to embrace AI applications in drug development, the market potential of AI in the pharmaceutical industry is expected to grow further. However, challenges remain, including data privacy concerns, the validation of AI models, and the integration of AI with existing drug development processes.

**Key Points**

This work highlights crucial aspects of drug discovery through three main contributions:

- A comprehensive overview of databases and tools frequently used in DTI and DTA prediction models,
- Evaluation of DTI and DTA prediction models with analysis of performance differentials,
- Case studies examining a large compound library against target proteins overrepresented in cancers.

## Data Availability

No new data were generated or analyzed in support of this research.
